# Li^+^ Conduction in a Polymer/Li_1.5_Al_0.5_Ge_1.5_(PO_4_)_3_ Solid Electrolyte and Li-Metal/Electrolyte Interface

**DOI:** 10.3390/molecules28248029

**Published:** 2023-12-10

**Authors:** Qinghui Li, Xiaofen Wang, Linlin Wang, Shyuan Zhu, Qingdong Zhong, Yuanyuan Li, Qiongyu Zhou

**Affiliations:** 1 School of Electrical & Information Engineering, Changsha University of Science & Technology, Changsha 410114, China; qinghuili@csust.edu.cn; 2 School of Materials Science and Hydrogen Energy, Foshan University, Foshan 528000, China; wangxiaofen@shu.edu.cn (X.W.); 20200580403@stu.fosu.edu.cn (L.W.); 2112206006@stu.fosu.edu.cn (S.Z.); liyuanyuan@wust.edu.cn (Y.L.); 3 School of Materials Science and Engineering, Shanghai University, Shanghai 200444, China

**Keywords:** composite solid electrolyte, polymer membrane, Li-ion conduction, interfacial transfer, NASICON

## Abstract

The solid oxide electrolyte Li_1.5_Al_0.5_Ge_1.5_(PO_4_)_3_ (LAGP) with a NASICON structure has a high bulk ionic conductivity of 10^−4^ S cm^−1^ at room temperature and good stability in the air because of the strong P^5+^-O^2−^ covalence bonding. However, the Ge^4+^ ions in LAGP are quickly reduced to Ge^3+^ on contact with the metallic lithium anode, and the LAGP ceramic has insufficient physical contact with the electrodes in all-solid-state batteries, which limits the large-scale application of the LAGP electrolyte in all-solid-state Li-metal batteries. Here, we prepared flexible PEO/LiTFSI/LAGP composite electrolytes, and the introduction of LAGP as a ceramic filler in polymer electrolytes increases the total ionic conductivity and the electrochemical stability of the composite electrolyte. Moreover, the flexible polymer shows good contact with the electrodes, resulting in a small interfacial resistance and stable cycling of all-solid-state Li-metal batteries. The influence of the external pressure and temperature on Li^+^ transfer across the Li/electrolyte interface is also investigated.

## 1. Introduction

Nowadays, the heavily used conventional vehicles have consumed large amounts of fossil fuels and caused severe environmental issues globally. To alleviate this problem, the governments of many countries continuously introduce incentive policies for the rapid development of pure electric vehicles (EVs) [1,2]. To date, the limited driving range of EVs, which causes range anxiety, still requires resolution. Therefore, the mass and volume energy density of the batteries keeps increasing to meet the demand of the application [3]. Lithium metal is considered the most promising anode for rechargeable Li-ion batteries due to its low density (0.59 g cm^−3^), high theoretical specific capacity (3860 mAh g^−1^), and its having the lowest working potential (−3.04 V vs. the standard hydrogen electrode) [4,5,6]. However, the metallic lithium anode reacts with the ether/ester based on an organic electrolyte and forms an uneven solid electrolyte interphase, resulting in the irregular deposition of lithium during charging and fast lithium dendrite formation. The continuous growth of lithium dendrites leads to capacity decay and severe safety problems (e.g., fire, explosion, etc.), and the consumption of organic electrolytes reduces the coulombic efficiency and cycling life of the rechargeable Li-metal batteries [7,8,9]. Therefore, it is practical to solve these problems by replacing the conventional organic liquid electrolyte with a fast Li^+^-conducting solid electrolyte having high mechanical strength [10,11,12].

During the past decade, research on solid electrolytes has been focused on different compositions and structures, including sulfide [13,14,15,16], oxide [17,18,19,20], halide [21,22,23], and composite electrolytes [24,25,26]. Sulfide and halide electrolytes have high room-temperature ionic conductivities above 10^−3^ S cm^−1^, but these solid electrolytes are moisture sensitive, and their ionic conductivities will significantly reduce in moist air. Oxide electrolytes have relatively high ionic conductivities from 10^−4^ to 10^−3^ S cm^−1^ at room temperature, and most oxide electrolytes are stable in air, which will reduce the handling cost of oxide electrolytes during cell preparation. Oxide electrolytes with garnet, NASICON, and perovskite structures have fast Li-ion transport channels and high Li-ion conductivity. Among them, the NASICON oxide with the general formula LiM_2_(PO_4_)_3_ (M = Ti and Ge, etc.) shows a high ionic conductivity of 10^−3^ S cm^−1^ at room temperature and good chemical stability in moist air because P^5+^-O^2−^ has a strong covalent bonding [27,28,29,30]; large-scale commercial production of Li_1.3_Al_0.3_Ti_1.7_(PO_4_)_3_ (LATP) and Li_1.5_Al_0.5_Ge_1.5_(PO_4_)_3_ (LAGP) has been achieved. However, applying LATP/LAGP electrolytes in all-solid-state Li-metal batteries still faces significant challenges. The poor solid–solid physical contact between LATP/LAGP ceramic and the metallic lithium anode leads to the large interfacial resistance of the battery, and the uneven local current distribution at the interface causes the formation and growth of lithium dendrites. Moreover, the Ti^4+^/Ge^4+^ ions of the LATP/LAGP electrolytes will be reduced on contact with lithium metal, which hinders the application of these electrolytes in rechargeable Li-metal batteries [31,32]. Therefore, it is urgent to develop solid organic/NASICON polymer electrolytes to solve the problems of NASICON electrolytes. 

Unlike rigid inorganic electrolytes, solid polymer electrolytes are flexible and have good contact with electrodes in all-solid-state Li-metal batteries. Moreover, the preparation process and the thickness of solid polymer electrolytes could be well controlled based on the technique of conventional Li-ion batteries; a thin and robust polymer electrolyte with a low density also enables the batteries with a high energy density. Polyethylene oxide (PEO), as a high Li^+^-conducting polymer electrolyte, has been widely investigated [33]; the dissolved lithium salts provide Li^+^ conduction through the motion of the polymer chain [34,35]. However, PEO polymer has a mixed amorphous and crystalline phase at room temperature [33,35]. Only the amorphous component can move freely to conduct Li^+^, and the crystalline PEO phase blocks the Li^+^ transport and reduces the ionic conductivity of the PEO electrolyte. 

Different strategies, including adding plasticizers/ceramic fillers and preparing crosslinked/block polymers, have been investigated to inhibit PEO crystallization and increase the concentration of the amorphous phase [36,37,38,39,40]. Among them, adding fillers to the polymer electrolyte is simple and effective in improving the Li^+^ conductivity and mechanical strength of the polymer electrolytes. Although different ceramic fillers, such as Li-ion conductors like garnet electrolytes and Li-ion insulators like Al_2_O_3_, have been investigated in polymer electrolytes, the ionic conductivity of polymer electrolytes is still not high enough for their application [37,41]. Moreover, the influence of external pressure and temperature on Li^+^ transport at the Li/polymer electrolyte interface is not well studied. Suitable pressure during cell testing helps solve the volume change problem, however, the cell could be short-circuited at a large pressure, and the cell will have high internal resistance at a small working pressure. Here, we introduce NASICON LAGP particles with high ionic conductivity to prepare PEO/LiTFSI/LAGP composite solid electrolytes. The results showed that adding LAGP particles increased the content of the PEO amorphous phase and effectively improved the ionic conductivity of the composite electrolyte. Moreover, the coating of LAGP particles with PEO prevented the reduction in LAGP by the metallic lithium anode. The PEO/LAGP composite solid electrolyte has a high ionic conductivity of 8 × 10^−5^ S cm^−1^ at room temperature and an increased electrochemical window of 4.5 V, which is much better than the PEO/LiTFSI electrolytes. The all-solid-state Li-metal battery with PEO/LiTFSI/LAGP electrolyte realized stable cycling at room temperature. The interfacial resistance of PEO/LAGP solid-state electrolytes and lithium metal can be significantly reduced with external pressure and high-temperature treatment.

## 2. Results and Discussion

**Figure 1:**
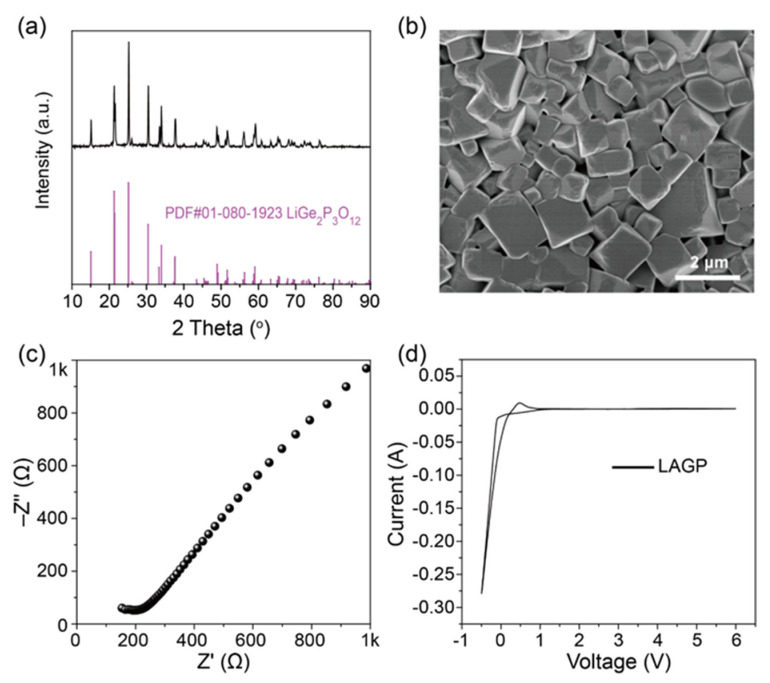
(**a**) XRD; (**b**) SEM; (**c**) impedance spectrum; and (**d**) CV curve of NASICON LAGP electrolyte after high-temperature sintering.

The XRD pattern of the LAGP pellet prepared using the solid-state reaction is shown in Figure 1a; all of the XRD peaks of the samples after high-temperature sintering agreed well with the standard NASICON LiGe_2_(PO_4_)_3_ (PDF#01-080-1923), confirming the high purity of the LAGP electrolyte. The SEM results of the LAGP powders in Figure 1b showed the LAGP particles have smooth surfaces and uniform size distribution (1–2 μm). In order to test the Li^+^ conductivity of the LAGP solid electrolyte, the Li^+^-blocking Ag electrode was evenly coated on both sides of the LAGP pellet, and Figure 1c shows the impedance spectrum of the LAGP pellet. The curve could be fitted with the equivalent circuit (R_b_//CPE/R_gb_//CPE); R_b_, R_gb_, and CPE are the bulk resistance, grain-boundary resistance, and constant phase components, respectively. The impedance spectrum has an incomplete depressed semicircle in the high-frequency region, corresponding to Li^+^ conduction at the grain boundary, and the bulk resistance is the intercept between the zero point and the starting point of the curve. The tail of the curve at the low-frequency region is caused by the Li^+^-blocking Ag electrode. The bulk and total ionic conductivity of the LAGP pellet are 4 × 10^−4^ and 2 × 10^−4^ S cm^−1^, respectively. The CV experiment of LAGP in Figure 1d was conducted to confirm the electrochemical stability of LAGP solid electrolytes; the LAGP solid electrolyte was electrochemically stable within a voltage window of 1–6 V, and the two peaks at 0.5 and −0.5V were mainly from the plating and stripping of lithium, respectively. However, the larger current density during the anodic process is possibly related to the reduction in Ge^4+^ and P^5+^ ions at low voltages. Therefore, preparing composite electrolytes by coating the surface of LAGP powders with PEO could help increase the stability of LAGP against lithium metal.

**Figure 2:**
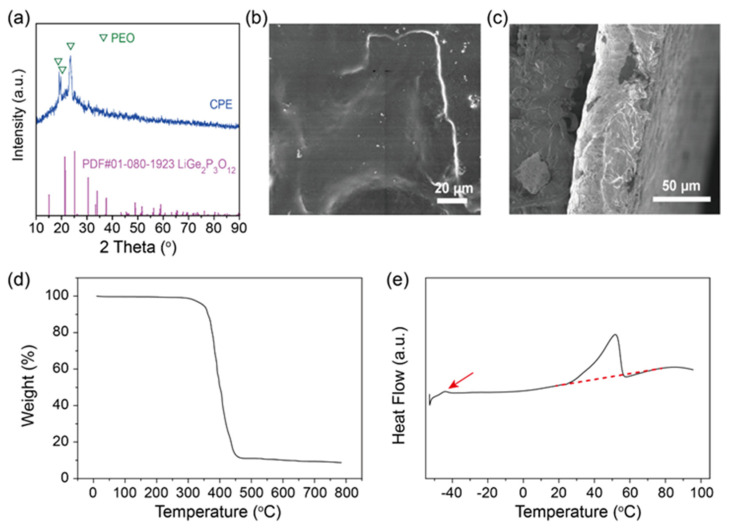
(**a**) XRD; (**b**) surface image; (**c**) cross-section image; (**d**) TGA; and (**e**) DSC curve of the PEO/LiTFSI/LAGP composite solid electrolyte.

The XRD pattern of the PEO/LiTFSI/LAGP composite solid electrolyte is shown in Figure 2. Due to the low concentration of LAGP in the polymer electrolyte, no diffraction peaks of LAGP were observed in the composite electrolytes. The diffraction peaks at 19 and 23 degrees in the composite electrolyte were from the crystalline PEO phase, and the broad hump from 20 to 25 degrees was from the amorphous PEO, confirming the coexistence of crystallization and the amorphous phase of PEO in the composite electrolyte. The crystalline PEO was significantly reduced compared to the PEO electrolyte without ceramic fillers. Figure 2b shows SEM images of the surface of the PEO/LiTFSI/LAGP composite electrolyte. The composite electrolyte has a relatively smooth surface and few closed pores; moreover, the LAGP particles are evenly dispersed in polymer electrolytes. The cross-section of the composite solid electrolyte (Figure 2c) shows that the membrane is dense and uniform with a thickness of 50 μm, and no pores are observed. Due to the brittleness of inorganic solid electrolytes, the thickness of inorganic solid electrolyte membranes like LAGP pellets in all-solid-state batteries is usually above 200 μm. In contrast, the smaller thickness of the PEO/LAGP composite solid electrolyte helps to realize a higher energy density of all-solid-state Li-metal batteries.

The residual solvent and adsorbed water in the composite solid electrolyte will affect the performance of the polymer electrolyte. A TGA experiment was employed to determine the thermal stability of the PEO/LiTFSI/LAGP solid electrolyte and whether there was residual solvent in the composite electrolyte; the result of the composite electrolyte is shown in Figure 2d, and there was almost no weight loss at temperatures below 300 °C. The thermal decomposition of PEO and lithium salts occurred from 300 to 450 °C. Moreover, there was almost no solvent and water absorption in the polymer electrolytes. The DSC curve of the PEO/LAGP composite electrolyte (Figure 2e) showed the membrane had a glass phase transition temperature of −42.3 °C and a melting temperature of 50 °C, indicating higher amorphous phase content of the composite electrolyte at room temperature, which agrees well with its higher Li^+^ conductivity.

**Figure 3:**
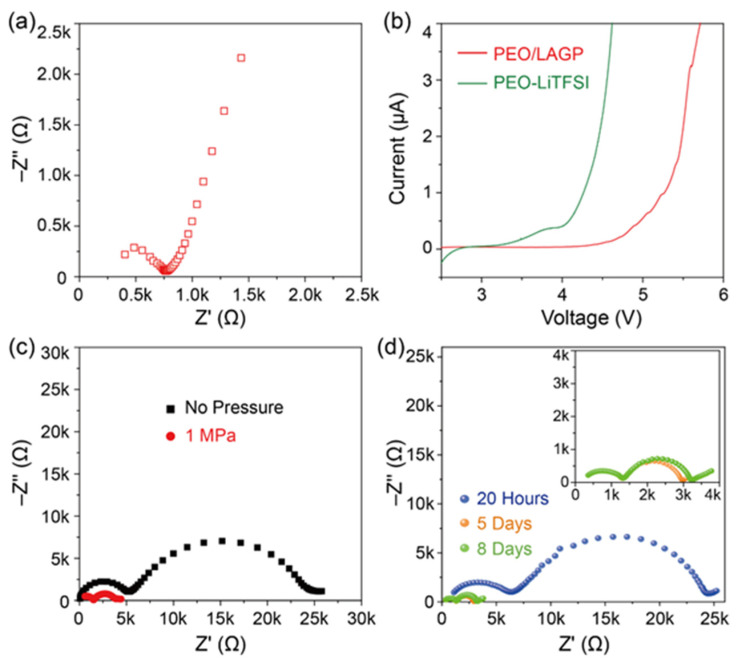
(**a**) The impedance spectra; (**b**) the LSV curve of the composite electrolytes of the membranes; (**c**) the impedance spectra of the symmetric Li/composite electrolyte/Li cell before and after applying an external pressure; and (**d**) heat treatment.

The impedance spectrum and fitting curve of the PEO/LiTFSI/LAGP composite solid electrolyte at room temperature are shown in Figure 3a. According to the fitting results, the Li^+^ conductivity of the composite solid electrolyte was 8 × 10^−5^ S cm^−1^ at 25 °C, which is one order of magnitude higher than that of the PEO/LiTFSI electrolyte; the addition of LAGP as a filler to PEO/LiTFSI can effectively improve the Li^+^-ion conductivity of polymer electrolytes.

The electrochemical stability of the composite electrolyte is an important indicator to determine the applied voltage range of the solid electrolyte in all-solid-state batteries. The LSV curves of the PEO/LiTFSI and PEO/LiTFSI/LAGP composite solid electrolytes are shown in Figure 3b. The PEO/LiTFSI electrolyte showed an increased oxidation current at 3.5 V; the narrow voltage window of the PEO/LiTFSI electrolytes limits their application in batteries with a high-voltage cathode. When the LAGP filler was added to the PEO/LiTFSI electrolyte, the electrochemical stability of the PEO/LAGP composite solid electrolyte was significantly improved, and the oxidation current of the cell began to increase at voltages above 4.5 V (Figure 3b). The increased stability may be related to the ceramic/PEO interaction, which is confirmed by several previous reports [41,42]. The cell had a small current less than 1 μA at 5.0 V vs. Li^+^/Li. The large electrochemical window of the PEO/LiTFSI/LAGP electrolytes enables their application in batteries with high-voltage cathodes like LiNi_0.8_Co_0.1_Mn_0.1_O_2_, which helps increase the energy density of the batteries.

In order to study the influence of applied external pressure on the interfacial resistance of the PEO/LAGP composite solid electrolyte and lithium metal, different external pressure was applied to the symmetric Li/Li cell, and the impedance spectra of the cells were measured, as shown in Figure 3c. The impedance spectra of the cell has a semicircle in the high-frequency and low-frequency regions, respectively; the low-frequency part represents the interfacial resistance, which can be attributed to the phase boundary resistance between organic polymers and ionic ceramics fillers or Li^+^ transport resistance through the Li anode/composite electrolyte interface [43,44]. The symmetric cell had a total impedance of 25,000 Ω with no external pressure. When 1 MPa pressure was applied to the Li/Li symmetric cell, the total impedance of the cell was reduced to 4000 Ω, which is only 1/6 of the total resistance of a cell without pressure; applying a certain pressure can greatly reduce the internal resistance. The resistance of the solid composite electrolyte and the Li/electrolyte interface of the cell with no pressure and 1 MPa pressure was obtained by fitting the impedance spectra. The resistance of the PEO/LAGP composite solid electrolyte decreased from 5031 to 1497 Ω at 1 MPa. The composite solid electrolyte has good characteristics of flexibility and ductility, and the external pressure could reduce the thickness of the electrolyte and increase the Li/electrolyte contact and Li^+^ transport at the interface. In addition, intimate interfacial contact between the phase boundary can be stimulated with appropriate external pressure, resulting in a decrease in interfacial resistance [45,46]. The interfacial resistance between the PEO/LAGP composite solid electrolyte and lithium metal was 1194 Ω at 1 MPa, which is only 1/8 of the cell with no pressure (9684 Ω). The cell short-circuited when the pressure applied to the Li/Li symmetric was further increased to 2 MPa. Due to excessive pressure, the flexible PEO/LAGP composite solid electrolyte becomes very thin, and the bumps or edges on the lithium metal sheet may puncture the electrolyte and cause a short circuit. Therefore, applying 1 MPa pressure to the Li/CPE/Li symmetric cell can effectively reduce the interfacial resistance.

The effect of heat treatment on the interfacial resistance between the composite electrolyte and lithium metal is rarely reported. In order to study the influence of temperature on the interface between the PEO/LAGP composite solid electrolyte and lithium metal, the assembled Li/Li symmetrical cell with the composite electrolyte was kept at 60 °C for different times. After the treatment, the total resistance of the symmetric cell was significantly reduced (Figure 3d). The impedance data of the symmetric Li/Li cell at room temperature were fitted to obtain the resistance of the solid electrolyte and the Li/electrolyte interface. After being held at 60 °C for 20 h, the resistances of the electrolyte and interface at room temperature were 7123 and 8493 Ω, respectively. After being held at 60 °C for 5 days, the resistance (1235 Ω) of the composite solid electrolyte at 25 °C was only 1/5 of that before the heat treatment, and the interfacial resistance was reduced to 876 Ω. The composite solid electrolyte had an interfacial resistance of 934 Ω after being held at 60 °C for 8 days. The heat treatment helps reduce the resistance of the electrolyte because high temperature will increase the amorphous phase of the polymer. The reduction in the Li/electrolyte interface could be related to better contact between polymer electrolytes and Li-metal at high temperatures. Moreover, the high temperature could help increase the wetting ability of the solid polymer electrolytes by the Li-metal anode.

**Figure 4:**
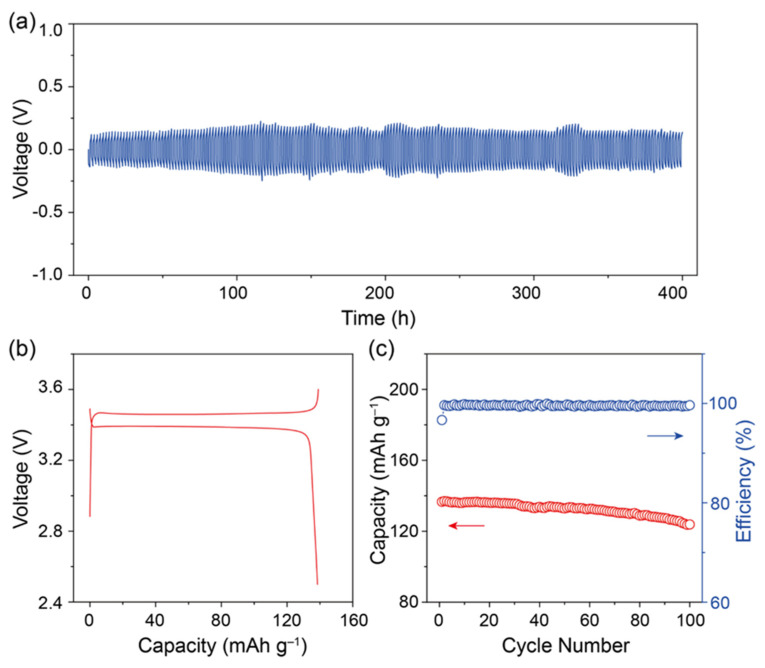
(**a**) The cycling performance of the symmetric Li/Li cell with the composite electrolyte; (**b**) the voltage/capacity curve, and (**c**) the cycling performance of the all-solid-state Li/LFP cell with the composite electrolyte at 25 °C.

The Li/Li symmetrical cell was held at 60 °C for 5 days and was cycled at a constant current (0.05 mA cm^−2^) at 25 °C. Figure 4a shows the voltage curve of the symmetric cell, and the cell had a stable polarization of 0.18 V and a long cycling life of more than 300 h. There was no voltage increase and no short-circuit of the symmetric cell, indicating that the cell had a stable electrolyte and Li/electrolyte interface. The testing temperature of all-solid-state Li-metal batteries in most reported results was from 40 to 60 °C, which limits their application at room temperature. Because the heat treatment (60 °C for 5 days) helped reduce the interfacial resistance, we assembled all-solid-state Li/LFP cells, and the cell was heated at 60 °C for 5 days and kept at 25 °C for 24 h. Figure 4b showed the charge/discharge curve of the all-solid-state Li/LFP cell from 2.5 to 3.6 V at 0.1 C and 40 °C, and the LFP cathode in the cell had a discharge capacity of 140 mAh g^−1^ at room temperature with an overpotential of 0.15 V. The cycling performance of the cell in Figure 4c showed more than 100 cycles at room temperature with a capacity retention of 85% and a relatively high coulombic efficiency of 99%. The stable cycling of the symmetric Li/Li cell and the Li/LFP cell confirmed the composite electrolyte could suppress lithium dendrite formation during cycling. Suitable external pressure and heating treatment could reduce the required working temperature for polymer-based solid batteries.

## 3. Materials and Methods

NASICON Li_1.5_Al_0.5_Ge_1.5_(PO_4_)_3_ (LAGP) powders were prepared using a conventional solid-state reaction. Li_2_CO_3_, (NH_4_)H_2_PO_4_, Al(OH)_3_, and GeO_2_ were ball-milled with a planetary ball-milling machine for 2 h; the materials were mixed and heated at 600 °C in the air in the box furnace for 6 h. The obtained powders were ball-milled for 3 h, pressed into pellets, and sintered at 900 °C in the air for 24 h. The LAGP powders were prepared by grinding the LAGP pellet with a high-energy ball-milling machine (Pulverisette7 premiumline, Idar-Oberstein, Germany). PEO (molecular weight 600,000) and LiTFSI were dissolved in anhydrous acetonitrile solvent, and the molar ratio of [EO] to Li^+^ was 10:1. After stirring the solution at 60 °C for 12 h, LAGP powder (10 wt%) was added to the acetonitrile solution and stirred for 24 h. The obtained solution was poured into a polytetrafluoron (PTFE) plate and dried under a vacuum at 40 °C for 24 h to obtain the PEO/LAGP composite solid electrolyte (CPE). The blank sample of PEO/LiTFSI polymer without LAGP filler was prepared using the same process.

The phase composition and microstructure of LAGP and PEO/LiTFSI/LAGP composite electrolytes were analyzed with a Rigaku MiniFlex 600 X-ray diffraction (XRD, Tokyo, Japan, Rigaku Corp.) apparatus and a FEI Quanta 650 scanning electron microscope (SEM, Eindhoven, North Brabant, The Netherlands), respectively. Ag and stainless steel were used as the blocking electrodes for LAGP and the composite polymer electrolytes, respectively, to confirm their ionic conductivity. The AC impedance spectra of the solid electrolytes were collected with the AutoLab workstation; the applied amplitude and frequency ranges were 10 mV and 1 MHz to 0.1 Hz, respectively. The cyclic voltammetry (CV) and the linear sweep voltammetry (LSV) curves of the LAGP and the composite electrolyte were collected with a scanning rate of 1 mV s^−1^; Li/LAGP/Au and Li/composite electrolyte/stainless steel cells were assembled for the CV and LSV measurement, respectively. The TG curves of the PEO/LiTFSI/LAGP composite electrolytes were measured with a Mettler thermogravimetric analyzer at a heating rate of 10 °C min^−1^ from 20 to 800 °C. The glass phase transition temperature and melting process of the composite electrolytes were determined using a Mettler-Toledo differential scanning calorimeter. 

The performance of the PEO/LiTFSI/LAGP composite electrolyte in all-solid-state Li-metal batteries was tested in Li/LiFePO_4_ and symmetric Li/Li batteries. LiFePO_4_ composite electrode is prepared by mixing LiFePO_4_, carbon, PEO, and LiTFSI salt (mass ratio: 70:10:13:7) in anhydrous acetonitrile solvent; the obtained slurry was coated on a carbon-coated Al current collector and vacuum dried at 60 °C for 24 h. The loading of LiFePO_4_ was 4 mg cm^−2^. The Li/Li symmetric and Li/LiFePO_4_ batteries were tested in the landing testing system; the testing voltage range of the Li/LiFePO_4_ cell was from 2.5 to 3.6 V.

## 4. Conclusions

A PEO/LiTFSI composite solid electrolyte with Li_1.5_Al_0.5_Ge_1.5_(PO_4_)_3_ (LAGP) as the ceramic filler was prepared, and the composite electrolyte has a high Li-ion conductivity of 8 × 10^−5^ S cm^−1^ at room temperature and good electrochemical stability above 4.5 V vs. Li^+^/Li. The influence of external pressure and temperature on the interfacial resistance of the Li/composite electrolyte was investigated in the symmetric Li/Li cell, and the optimized pressure and heat treatment were 1 MPa and 60 °C for 5 days, respectively, which reduced the interfacial resistance to 1/8 and 1/10 of that measured at room temperature. The symmetric Li/Li cell had a long cycling life of 320 h at room temperature, indicating the good capability of the composite electrolyte to suppress dendrite formation and growth. Moreover, the all-solid-state Li/LFP battery had a high discharge capacity of 140 mAh g^−1^ and relatively stable cycling at 40 °C.

## Data Availability

The data presented in this study are available on request from the corresponding author. The data are not publicly available due to [containing commercially sensitive information].
